# DNA Damage Enhanced by the Attenuation of SLD5 Delays Cell Cycle Restoration in Normal Cells but Not in Cancer Cells

**DOI:** 10.1371/journal.pone.0110483

**Published:** 2014-10-21

**Authors:** Zhi-Yuan Gong, Hiroyasu Kidoya, Tomomi Mohri, Yinglu Han, Nobuyuki Takakura

**Affiliations:** 1 Department of Signal Transduction, Research Institute for Microbial Diseases, Osaka University, Suita, Osaka, Japan; 2 Japan Science and Technology Agency, Chiyoda-ku, Tokyo, Japan; Osaka University Graduate School of Medicine, Japan

## Abstract

SLD5 is a member of the GINS complex composed of PSF1, PSF2, PSF3 and SLD5, playing a critical role in the formation of the DNA replication fork with CDC45 in yeast. Previously, we had isolated a PSF1 orthologue from a murine hematopoietic stem cell DNA library and were then able to identify orthologues of all the other GINS members by the yeast two hybrid approach using PSF1 as the bait. These GINS orthologues may also function in DNA replication in mammalian cells because they form tetrameric complexes as observed in yeast, and gene deletion mutants of both PSF1 and SLD5 result in a lack of epiblast proliferation and early embryonic lethality. However, we found that PSF1 is also involved in chromosomal segregation in M phase, consistent with recent suggestions that homologues of genes associated with DNA replication in lower organisms also regulate cellular events other than DNA replication in mammalian cells. Here we analyzed the function of SLD5 other than DNA replication and found that it is active in DNA damage and repair. Attenuation of SLD5 expression results in marked DNA damage in both normal cells and cancer cells, suggesting that it protects against DNA damage. Attenuation of SLD5 delays the DNA repair response and cell cycle restoration in normal cells but not in cancer cells. These findings suggest that SLD5 might represent a therapeutic target molecule acting at the level of tumor stromal cells rather than the cancerous cells themselves, because development of the tumor microenvironment could be delayed or disrupted by the suppression of its expression in the normal cell types within the tumor.

## Introduction

Cells are constantly exposed to genomic DNA damage caused by internal and external agents such as oxidative stress and UV, respectively. Errors in DNA damage repair can result in cancer cell development [1], [2]. To prevent oncogenic transformation, normal cells monitor and repair DNA damage in their genome by setting cell cycle checkpoints [3]. However, cancer cells are able to tolerate DNA damage such that replication continues without repair, resulting in the accumulation of abnormal mutant gene expression [4]. This event has been suggested as one of the causes of chemo- and radio-resistance development in malignant cancer cells.

SLD5 is a member of the GINS complex composed of PSF1, PSF2, and PSF3. This complex regulates the DNA replication fork in budding yeast [5]. In the initiation of DNA replication, the origin recognition complex (ORC) binds to the autonomously replicating sequence (ARS) that functions as a DNA replication start domain. Subsequently, cell division cycle (Cdc) 6 and Cdc1 bind to ARS guided by ORC and induce binding of mini-chromosome maintenance (Mcm) proteins onto ARS. These are termed pre-replication complexes (pre-RC) [6]–[8]. Further, Cdc45 and GINS are recruited to pre-RC and form activated CMG (Cdc45-Mcm-GINS) helicase at the DNA replication fork [9]–[12].

We identified a mouse orthologue of PSF1 in a DNA library derived from hematopoietic stem cells during embryogenesis in which this cell population actively proliferates [13]. Subsequently, we identified SLD5 using a yeast two-hybrid system with PSF1 as the bait [14]. Moreover, we identified all members of GINS in mice and confirmed that they form complexes as observed in yeast [15]. We previously reported that mutant mice deficient for PSF1 or SLD5 show early embryonic lethality caused by the growth arrest of epiblasts at embryonic day 6.5 [13], [16]. These findings suggested that PSF1 and SLD5 are functional in mammals and essential for cell proliferation, possibly associating with DNA replication as observed in yeast.

High expression of GINS genes has been observed in cancers and a correlation of their level of expression with malignancy has been suggested [17]–[19]. We also reported that cancer cells showing higher PSF1 promoter activity are cancer initiating/stem cells in a murine tumor cell transplantation model [20]. A feature of malignant cancer cells is chemo- and radio-resistance. High level expression of GINS genes may induce not only cell growth but also resistance to chemotherapy. However, it has not been determined whether the function of GINS genes is involved in DNA damage or repair. By observing bone marrow cellularity in mutant mice, we previously found that haploinsufficiency of PSF1, but not SLD5, reduces cell growth [13], [16]. Therefore, it is complicated to analyse the function of PSF1 in DNA damage by knocking down PSF1 expression because cell growth itself is also affected by lack of this factor. In case of SLD5, heterozygous SLD5^+/−^mice, which were healthy and fertile, were born at Mendelian frequency and exhibited normal growth. Moreover, there is no large difference of bone marrow cellularity between wild and SLD5^+/−^ mice [16]. Therefore, we used SLD5^+/−^ mouse embryonic fibroblasts (MEFs) to analyze DNA damage repair and cell growth after DNA damage. Moreover, we compared the function of SLD5 in DNA damage repair using siRNA knock-down experiments in cancer cells.

## Materials and Methods

### Cell culture and drug treatment

MEFs, B16 cells (mouse melanoma cells), and colon26 cells (mouse colon cancer cells) were grown in Dulbecco's modified Eagle's medium (DMEM) (Sigma) with 10% fetal bovine serum (FBS; Sigma), and penicillin/streptomycin (Sigma) at 37°C under an atmosphere of 5% CO_2_. MEFs were prepared from wild-type (WT) or SLD5^+/−^ mice at embryonic day (E) 15.5 according to the usual method [16]. B16 cells and colon26 cells were purchased from the Riken cell bank (Tsukuba, Japan). Cells were treated with 1 µM or 10 µM etoposide (Sigma). To induce DNA damage strongly, we used 10 µM etoposide. However, 10 µM etoposide severely induced cell apoptosis. Therefore, we used 1 µM etoposide to observe cell cycle restoration. Animals were housed in environmentally-controlled rooms of the animal experimentation facility at Osaka University. All experiments were conducted under the applicable laws and guidelines for the care and use of laboratory animals in the Research Institute for Microbial Diseases, Osaka University, approved by the Animal Experiment Committee of the Research Institute for Microbial Disease, Osaka University (Permit number 3239–6). All surgery was performed under sodium pentobarbital anesthesia, and all efforts were made to minimize suffering.

### siRNA transfection

SLD5 expression in B16 and Colon26 cells was transiently knocked down with small interfering RNA (siRNA). Lipofectamine RNAiMAX (Invitrogen) was used for the transfection of plasmid and siRNA into cells, following the manufactureŕs protocols, and experiments were done 48 h after transfection. We used two different siRNA oligonucleotides specific for SLD5; target siRNA sequences were: 5′-GGA CCA CAC GGA GAC CCA CUU UAA A-3′ (#1); 5′-GAU GAG CAG AGA GAC UAC GUG AUU G-3′ (#2).

### Trypan blue exclusion test for cell viability and regrowth after treatment with etoposide

5×10^3^ cells were seeded into each well of a 24-well culture plate (BD Falcon) in 500 µl of medium. After 24 h, the wells were exposed to 1 µM etoposide for 12 h. After the drug was removed, cells were harvested immediately (0 h). Cell viability was evaluated by trypan blue exclusion. The same number of living cells was resuspended in fresh medium, and cells were cultured for 24, 48 or 72 h. They were then detached by adding 100 µl Trypsin-EDTA to each well; cells were then washed and re-suspended in 400 µl of medium. 20 µl of re-suspended cells were mixed with 20 µl of 0.4% solution of trypan blue dye (Life Technologies) for 1 min. Cells were immediately counted using a Neubauer microchamber with a light microscope. All counts were done using four technical duplicates of each sample. Means and standard deviations were calculated for each subculture.

### Western blotting

Cells were collected and lysed in SDS sample buffer (50 mM Tris-HCL, pH 6.8, 2% sodium dodecyl sulfate, 6% 2-mercaptoethanol, 10% glycerol, 0.003% bromophenol blue) containing a cocktail of protease inhibitors. Proteins were separated on 10% or 15% SDS-PAGE and transferred onto PVDF membranes. After blocking for 1 h in TBST (25 mM Tris, pH 7.5, 1.37 M NaCl, 27 mM KCl, 0.05% Tween20) containing 2% non-fat dry milk, membranes were incubated with anti-SLD5 (1∶1000; Iwaki), anti-γ-H2AX (1∶500; Cell Signaling), anti-Rad51 (H-92; 1∶1000; Santa Cruz), or mouse anti-β-actin antibody in blocking buffer overnight. Membranes were then washed with TBST and incubated with HRP-conjugated anti-rat, rabbit or goat antibodies (1∶10000) for 1 h. Bound antibodies were detected with ECL kit**s** (Amersham). The immunoreactive proteins were visualized using the ECL Prime Western Blotting Detection system (GE Healthcare, Buckinghamshire). The blots were scanned using the imaging densitometer Las-300 mini (Fujifilm, Tokyo, Japan).

### Statistical Analysis

Results are expressed as the mean ± SD. Student's *t* test was used for statistical analysis. Differences were considered statistically significant when *p*<0.05.

## Results

### Enhanced DNA damage by the Attenuation of SLD5 expression in MEFs

**Figure 1:**
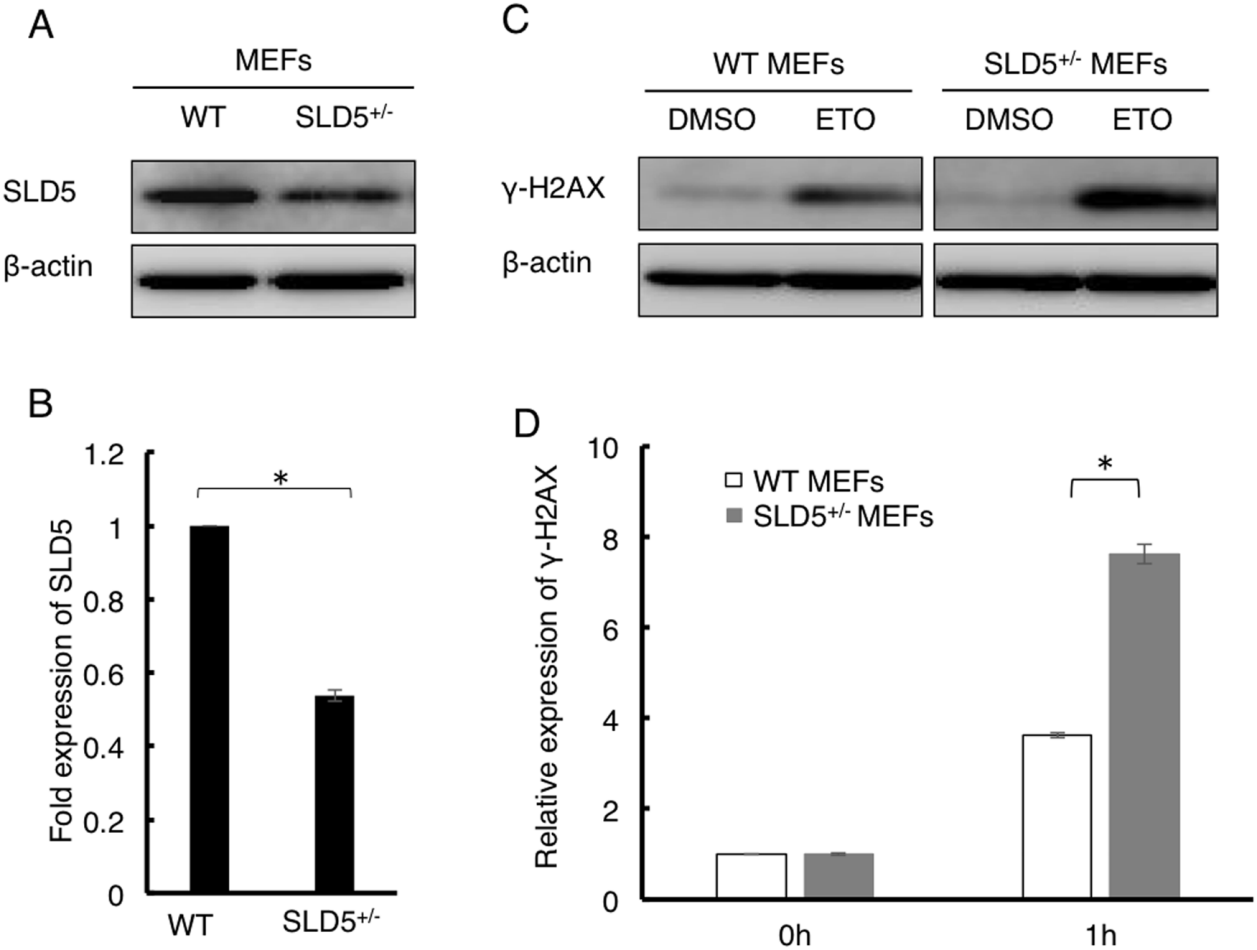
Attenuation of SLD5 expression results in marked DNA damage in MEFs. (**A**) Western blot analysis of SLD5 expression in WT and SLD5^+/−^ MEFs. β-actin was the internal control. (**B**) Quantitative evaluation of SLD5 expression as revealed in (**A**) based on densitometric analysis. Results are represented as fold-change compared with the level seen in WT MEFs. Data represent the mean ± SD. *, *P*<0.05 (n = 3). (**C**) Western blot analysis of γ-H2AX expression in WT and SLD5^+/−^ MEFs after treatment with etoposide (ETO) or control vehicle (DMSO). β-actin was the internal control. (**D**) Quantitative evaluation of γ-H2AX expression as revealed in (**C**). Results are fold-changes compared with the level seen in WT MEFs treated with DMSO. Data represent the mean ± SD. *, *P*<0.05 (n = 3).

To assess whether SLD5 relates to the DNA damage response, we compared the difference between DNA damage using MEFs from WT mice and SLD5^+/−^ mice [16]. We confirmed that the level of SLD5 in SLD5^+/−^ MEFs was approximately half of that in WT MEFs (**Figure 1A and B**). To investigate DNA damage, we challenged MEFs with etoposide, a topoisomerase Π inhibitor that induces DNA double-strand breaks [21], [22]. Cells were treated with 0.01% DMSO as a control or with 10 µM etoposide for 1 h. In the steady state (exposure to DMSO), the level of phosphorylation of the chromatin-bound histone H2AX (γ-H2AX), which is a quantitative marker for the DNA damage response at the site of double-strand breaks [23], [24], was similarly low in both WT and SLD5^+/−^ MEFs (**Figure 1C and D**). Exposure to etoposide led to an increase in the level of γ-H2AX in both WT and SLD5^+/−^ MEFs, but significantly more so in the latter (**Figure 1C and D**).

### Delay of cell cycle restoration by the attenuation of SLD5 expression in MEFs after DNA damage

**Figure 2:**
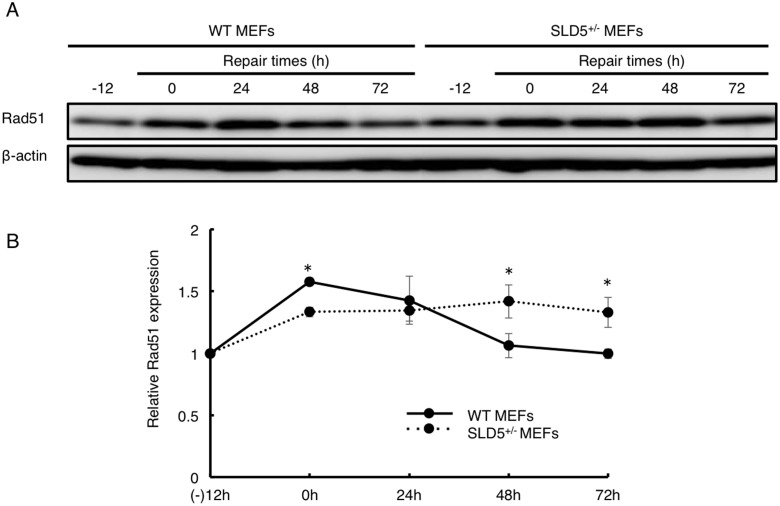
Delayed and prolonged Rad51 expression after DNA damage by etoposide in SLD5^+/−^ MEFs. (**A**) Western blot analysis of Rad51 expression in WT and SLD5^+/−^ MEFs. β-actin was the internal control. MEFs were treated with etoposide for 12 h (−12∼0) and lysed at the indicated times. (**B**) Quantitative evaluation of Rad51 expression as revealed in (**A**) based on densitometric analysis. Results are fold-change compared with the level seen in WT MEFs before treatment with etoposide. Data represent the mean ± SD.*, *P*<0.05 (n = 3).

**Figure 3:**
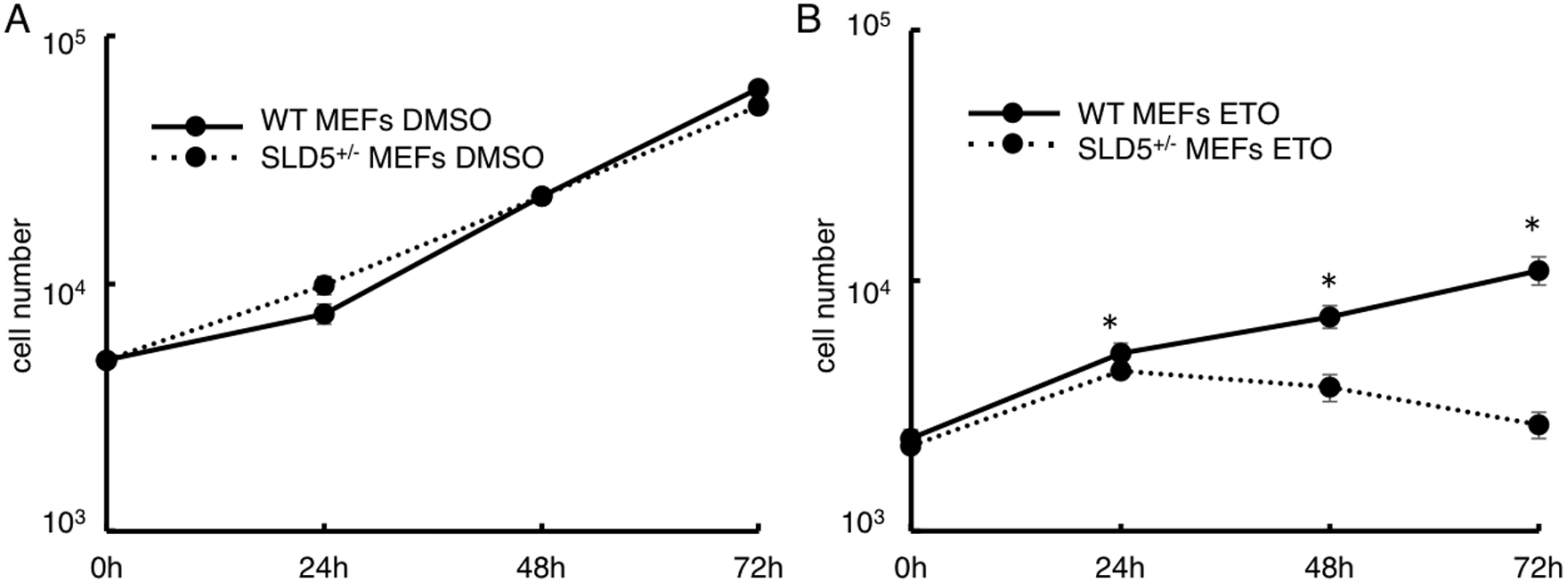
Impaired cell cycle restoration after DNA damage in SLD5^+/−^ MEFs. MEFs were treated with DMSO (**A**) or etoposide (**B**) as indicated in Figure 2 and the same number of living cells as indicated were cultured with fresh medium for 72 h. Cells were counted at the indicated time. Data represent the mean ± SD. *, *P*<0.05 (n = 3).

To elucidate whether marked DNA damage caused by the attenuation of SLD5 expression relates to DNA damage repair, we measured the level of Rad51 protein, which is the key component for homologous recombination [25]. As described above, MEFs were treated with 1 µM etoposide for 12 h, and Rad51 protein expression was then analyzed at 0, 24, 48 and 72 h after its removal (**Figure 2A, B**). The level of Rad51 expression was equivalent in WT and SLD5^+/−^ MEFs before treatment with etoposide. In WT MEFs, the level of Rad51 significantly increased rapidly after exposure to etoposide but less so in SLD5^+/−^ MEFs, and gradually decreased at 24, 48 h, almost returning to baseline at 72 h. In contrast, Rad51 protein in SLD5^+/−^ MEFs increased more slowly than in WT MEFs and was maintained for a longer time. This suggests that a longer period is required for DNA repair after extensive DNA damage in SLD5^+/−^ MEFs. Prolonged DNA repair time in SLD5^+/−^ MEFs in turn suggests delayed cell cycle restoration after DNA damage. Therefore, we counted the number of viable cells after treatment with etoposide as described above. Cell proliferation was determined by the trypan blue exclusion test. There was no significant difference between WT and SLD5^+/−^ MEF proliferation after exposure to the DMSO control (**Figure 3A**), suggesting that halved SLD5 expression in MEFs does not affect cell growth itself. By contrast, upon exposure to etoposide, SLD5^+/−^ MEFs displayed significantly retarded cell growth compared with WT MEFs (**Figure 3B**).

### DNA damage is enhanced in cancer cells by the attenuation of SLD5 expression

**Figure 4:**
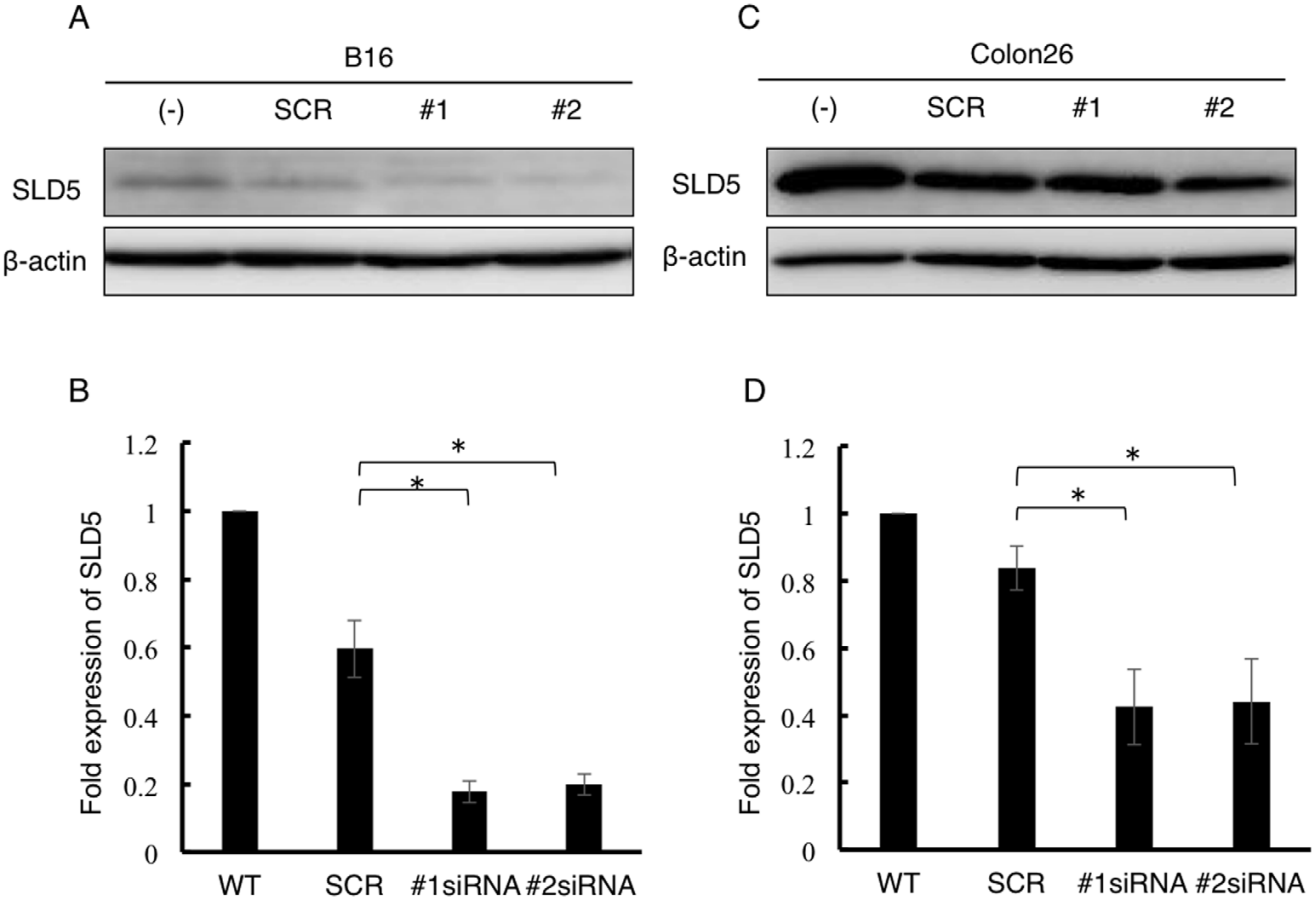
Silencing of SLD5 by siRNA in B16 and Colon26 cancer cells. B16 or Colon26 cells were transfected with scrambled negative control (SCR) or SLD5 siRNAs (#1, #2) and harvested 48 h after transfection. SLD5 protein expression levels in B16 (**A, B**) or Colon26 (**C, D**) were quantified by Western blotting. β-actin was the internal control. Data were quantitatively evaluated based on densitometric analysis (**B, D**). Results are represented as fold-change compared with the level seen in siRNA-untreated B16 cells (**B**) or Colon26 cells (**D**), respectively. Data represent the mean ± SD.*, *P*<0.05 (n = 3).

**Figure 5:**
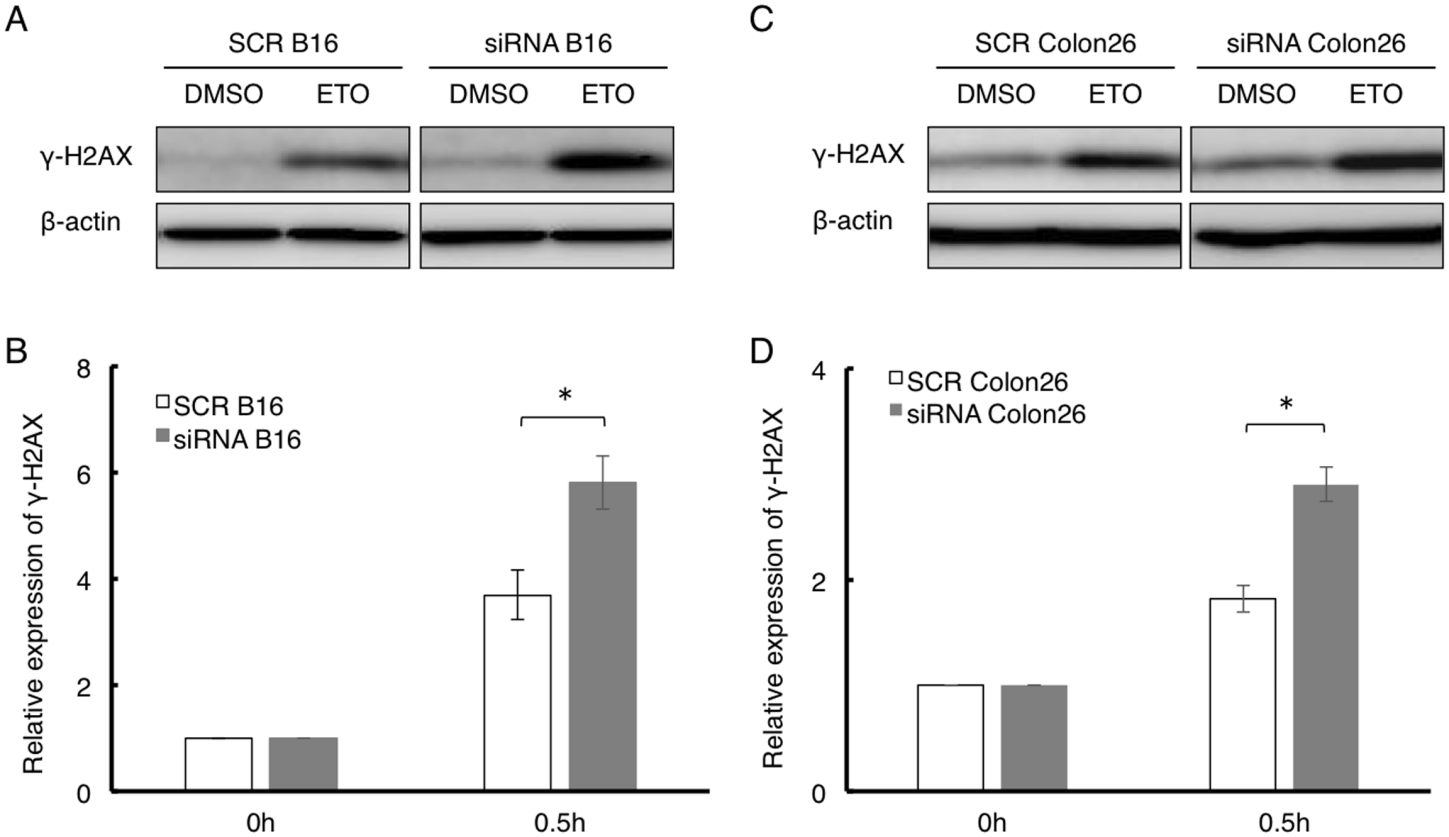
Attenuation of SLD5 expression induces marked DNA damage in cancer cells. Western blot analysis of γ-H2AX expression in SCR or SLD5 siRNA-transfected B16 (**A,** **B**) or Colon26 (**C,** **D**) cells after treatment with etoposide (ETO) or control vehicle (DMSO). β-actin was the internal control. Data were quantitatively evaluated based on densitometric analysis (**B**, **D**). Results are fold-change compared with the level seen in SCR siRNA-treated B16 cells (**B**) or Colon26 cells (**D**), respectively. Data represent the mean ± SD. *, *P*<0.05 (n = 3).

In normal cells such as MEFs, DNA damage was strongly induced by the attenuation of SLD5 expression. We next tested whether cancer cells also show similar responses to etoposide when SLD5 expression was prevented. To this end, SLD5 expression was knocked down in B16 mouse melanoma cells and Colon26 mouse colon cancer cells by transfecting siRNA (#1 and #2) directed against SLD5 (siRNA B16 and siRNA Colon26). We confirmed that SLD5 expression could be efficiently silenced by specific siRNA in B16 and Colon26 cells but not by scrambled control siRNA (SCR B16 and SCR Colon26) (**Figure 4A–D**). Using these cells, we quantified DNA damage by measuring the level of γ-H2AX by Western blotting. After maintenance in complete medium for 48 h, cells were treated with DMSO as a control or 10 µM etoposide for 0.5 h. The initial γ-H2AX expression level was not high in either SCR B16 or siRNA B16 cells (**Figure 5A, B**). Exposure to etoposide led to an increased level of γ-H2AX in both SCR B16 cells and siRNA B16 cells, but it was significantly higher in the latter (**Figure 5A, B**). Similarly, in Colon26 cells, attenuation of SLD5 expression enhanced DNA damage by etoposide (**Figure 5C, D**). Taken together, we conclude that SLD5 expression relates to DNA damage in normal cells and cancer cells.

### Attenuation of SLD5 expression in cancer cells does not delay cell cycle restoration after DNA damage

**Figure 6:**
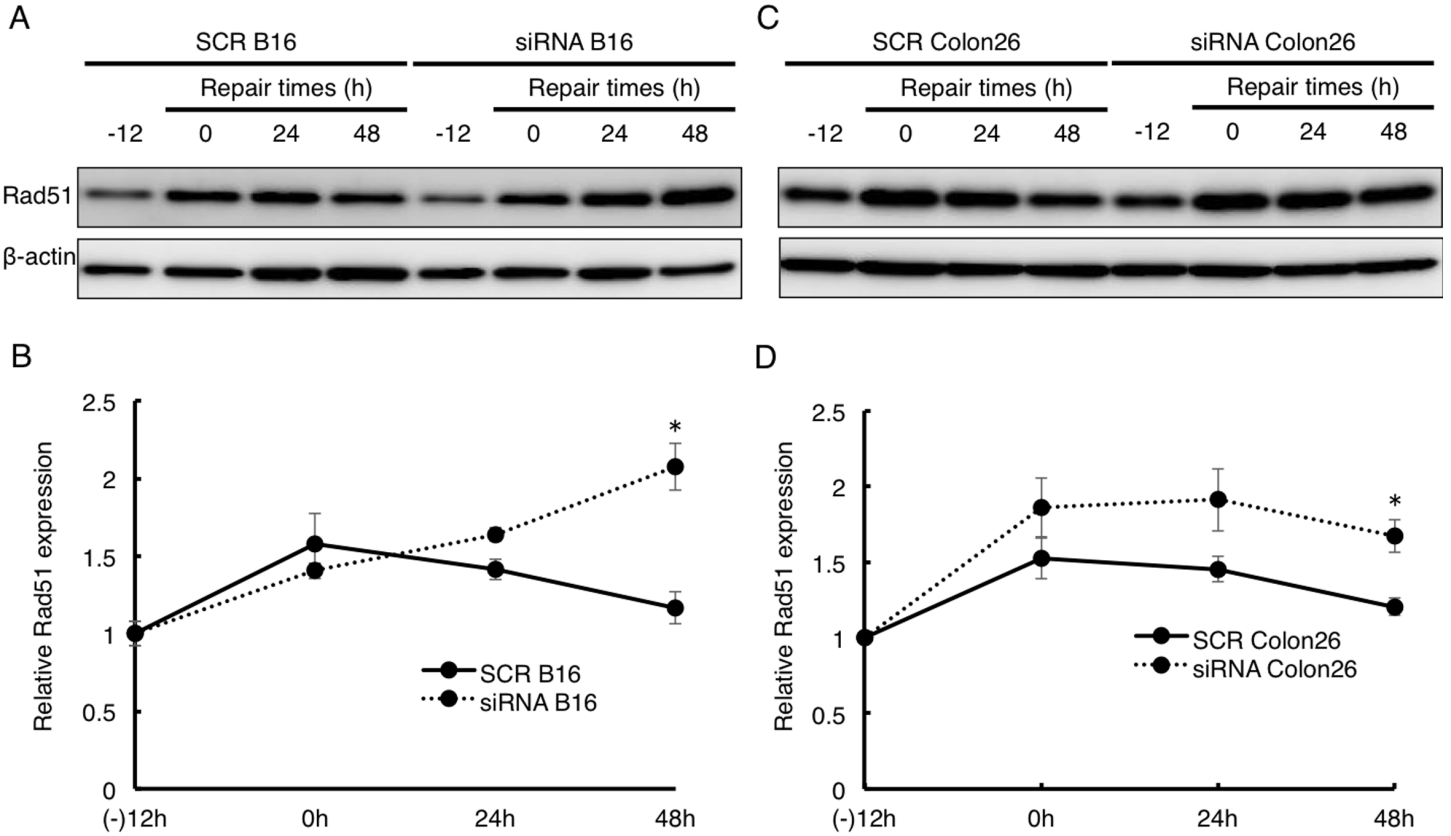
Prolonged Rad51 expression after DNA damage by etpoposide in cancer cells. SCR or SLD5 siRNA transfected B16 (**A, B**) or Colon26 (**C, D**) cells were treated with etoposide for 12 h (−12∼0). Cells were lysed at the indicated times. Western blot analyses of Rad51 expression is shown. β-actin was the internal control. Data were quantitatively evaluated based on densitometric analysis. Results are fold-change compared with the level seen in SCR B16 (**B**) or SCR Colon26 (**D**) before treatment with etoposide. Data represent the mean ± SD.*, *P*<0.05 (n = 3).

We predicted that attenuation of SLD5 expression would delay DNA repair and cell cycle restoration in cancer cells as observed in normal cells. However, although the level of Rad51 expression was the same in SCR B16 and SLD5 siRNA B16 cells, and in SCR Colon26 and SLD5 siRNA Colon26 cells before treatment with etoposide, thereafter, a rapid increase of Rad51 was similarly observed in all four cell lines (**Figure 6A–D**). Corresponding to the marked DNA damage in siRNA B16 and siRNA Colon26 cells, prolonged high level Rad51 expression was observed in these lines. Thus, an extended period for DNA repair was common to normal cells and cancer cells, but the diminished rapid response to DNA damage resulting from attenuation of SLD5 expression in normal cells was not seen in cancer cells.

**Figure 7:**
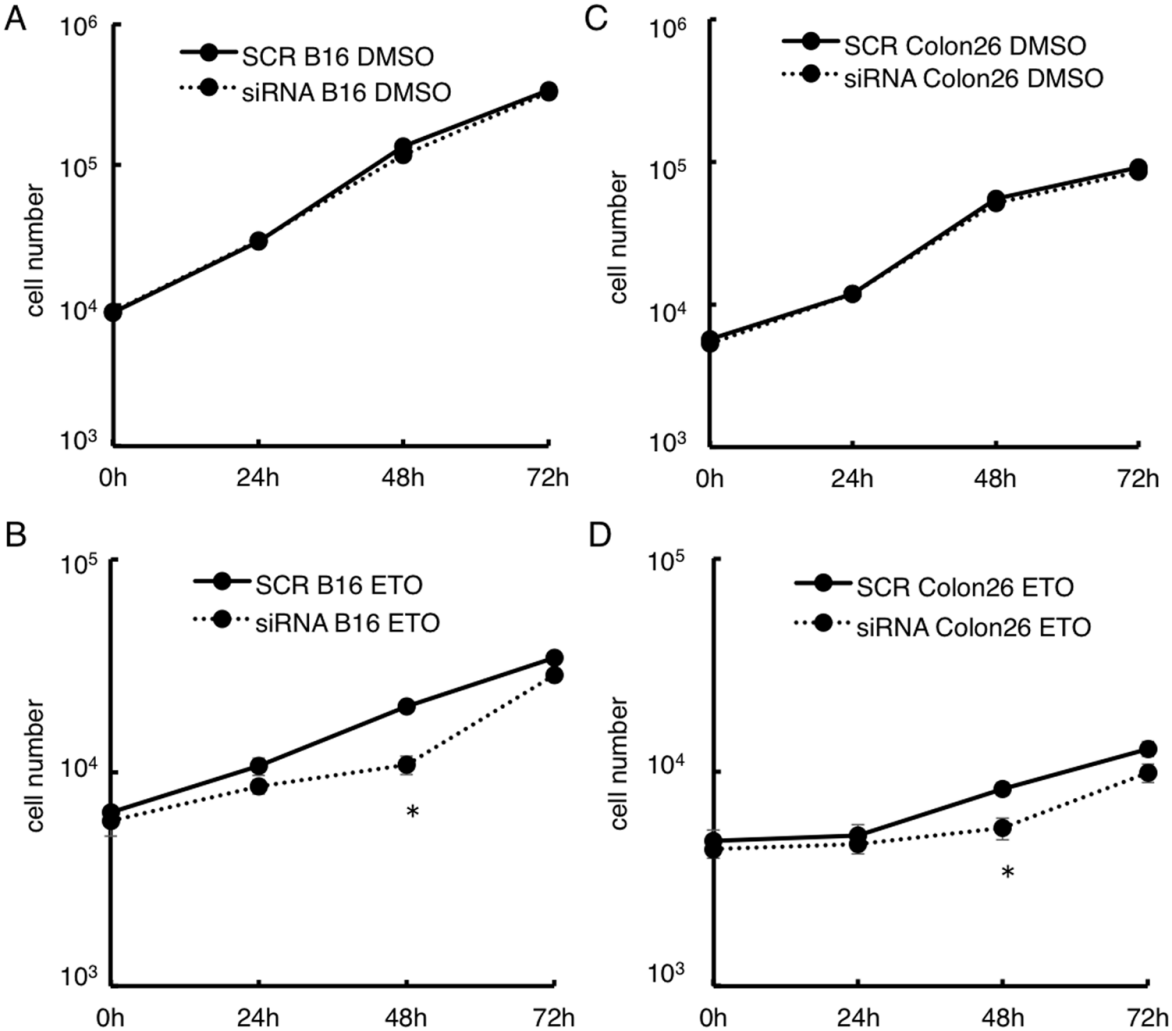
Cell cycle restoration after DNA damage in cancer cells. SCR or SLD5 siRNA transfected B16 (**A, B**) or Colon26 (**C, D**) cells were treated with DMSO (**A,** **C**) or etoposide (**B,** **D**) as indicated in Figure 6 and the same number of living cells as indicated were cultured with fresh medium for 72 h. Cell numbers were recorded. Data represent the mean ± SD.*, *P*<0.05 (n = 3).

To assess how the rapid response to DNA damage in SLD5 knocked-down cancer cells affects cell cycle restoration, we enumerated the cells after treatment with etoposide. Cell proliferation itself was not affected by knocking down SLD5 in either siRNA B16 or siRNA Colon26 cells in the presence of control DMSO treatment (**Figure 7A, C**). Forty-eight hours after etoposide-mediated DNA damage, there was only slight retardation of cell cycle restoration in both siRNA B16 and siRNA Colon26 relative to that seen in SCR B16 and SCR Colon26. However, after 72 h, cell growth was induced in SLD5-silenced cancer cells to the same extent as in controls in both B16 and Colon26 cells (**Figure 7B, D**). Therefore, we conclude that extensive DNA damage resulting from the attenuation of SLD5 expression severely affects cell cycle restoration in normal but not cancer cells.

## Discussion

It has been reported that SLD5 forms a GINS complex with other moieties such as PSF1, PSF2, and PSF3 and is involved in DNA replication in yeast [5]. In the present report, we propose that SLD5 is involved in DNA damage and repair in mammalian cells. Attenuation of SLD5 expression resulted in marked DNA damage by agents promoting DNA double strand breaks; this was common to normal cells and cancer cells. A longer period was required for restoration of the cell cycle when DNA damage was extensive. For this response, cells need to enhance DNA repair. When SLD5 expression was reduced in normal cells, expression of Rad51 was delayed, suggesting that SLD5 is also involved in DNA repair protein assembly. In contrast, rapid Rad51 expression was induced in cancer cells after DNA damage and delay of cell cycle restoration was not as severe as in normal cells. Roles of GINS genes in other types of cancers have been reported [17], [26]. Therefore, it suggests that the function of SLD5 in DNA damage and repair is also utilized in other tumor cell types than melanoma and colon cancer cells used in our experiments. Further experiments are required to clarify this.

As described above, the GINS complex is involved in DNA replication; however, GINS components PSF1, PSF2, PSF3, and SLD5 do not always form complexes and may have other functions. For instance, PSF1 regulates microtubule organization in M phase and is involved in chromosome segregation [20]. Recently, it has been reported that homologous molecules associating with DNA replication in lower organisms may also regulate cellular events other than DNA replication [27]–[29]. Therefore, it is possible that SLD5, a molecule involved in DNA replication in yeast, is also involved in DNA damage repair. A previous report suggested that DNA damage is prevented when chromatin is condensed with histone [30]. However, during DNA replication, naked DNA is dissociated from histone and is endangered by agents which induce DNA double strand breaks. How SLD5 protects from DNA double strand breaks is thus far unclear, but it may be involved in histone modification. Further precise analysis is required to elucidate the mechanism of DNA damage protection afforded by SLD5.

It was to be expected that a longer period of time would be required for DNA repair in cells with worse DNA damage as a result of lack of SLD5. We hypothesized that rapid Rad51 expression would be induced in SLD5^+/−^ MEFs compared with WT MEFs for repairing heavily damaged DNA. However, SLD5 attenuation was found to delay Rad51 expression in MEFs, resulting in severe retardation of cell cycle restoration. These findings suggested that SLD5 not only protects against DNA damage but regulates the rapidity of DNA repair. Recently, it has been reported that PSF2, also a member of the GINS complex, is phosphorylated by ATM upon DNA strand breakage [31]. Therefore, it is possible that PSF2 is involved in DNA repair too. Furthermore, it has been reported that the N-terminal and C-terminal domains of Sld5 interact with the N-terminal and the C-terminal regions of Psf2 in Crystal structure of the human GINS complex [32], and Sld5 was found to interact by two-hybrid with PSF2 in *Drosophila* [33]. It will be interesting to analyze interactions between SLD5 and phosphorylated PSF2 during DNA repair.

Recent studies have shown that tumor growth and metastasis are not determined by cancer cells alone but also by various stromal cells. The stroma constitutes a large part of most solid tumors, and the cancer-stromal cell interaction contributes functionally to tumor growth and metastasis [34], [35]. Tumor stroma contains many different types of cells, including cancer-associated fibroblasts (CAF), pericytes, endothelial cells, infiltrated immune cells. Among them, CAFs are the major cell type that play a crucial role in tumorigenesis and metastasis [36], [37]. The results of our experiments have shown that attenuation of SLD5 induced marked DNA damage and suppressed rapid cell cycle restoration in MEFs. Therefore, we predicted that SLD5 inhibitors could be candidate anti-cancer drugs. We found that in cancer cells, DNA damage is strongly induced by silencing SLD5 expression, as observed in MEFs; however, Rad51 rapidly increased and cell cycle restoration was not greatly affected. This suggests that cancer cells possess additional DNA repair machinery, independent of SLD5, and thereby maintain proliferative capacity. Silencing SLD5 in cancer cells may therefore not be effective as a strategy to inhibit tumor growth. However, not only cancer cells but also normal cell types such as endothelial cells and fibroblasts proliferate in the tumor microenvironment support growth of the tumor as stromal cell components [38]–[40]. Blocking angiogenesis has been shown to be an effective strategy in inhibiting tumor growth and metastasis [41]. Therefore, silencing SLD5 expression or suppression of SLD5 function by a small compound or a nucleoside analogue such as microRNA in tumor stromal cells may inhibit the development of the tumor microenvironment and could be a promising approach to inhibit tumor growth similar to the strategy of inhibiting tumor angiogenesis.
